# Warm intranasal saline irrigation reduces intraoperative blood loss during functional endoscopic sinus surgery: a propensity score-matched cohort study

**DOI:** 10.1080/07853890.2026.2690712

**Published:** 2026-06-22

**Authors:** Yung-Fong Tsai, Yu Chang, Sheng-Dean Luo, Amina M. Illias, Shao-Chun Wu

**Affiliations:** a Department of Anaesthesiology, Linkou Chang Gung Memorial Hospital, Taoyuan, Taiwan; b College of Medicine, Chang Gung University, Taoyuan, Taiwan; c Department of Anaesthesiology, Kaohsiung Chang Gung Memorial Hospital, Kaohsiung, Taiwan; d Department of Otolaryngology, Kaohsiung Chang Gung Memorial Hospital, Kaohsiung, Taiwan

**Keywords:** Functional endoscopic sinus surgery, irrigation temperature, blood loss, propensity score matching, bispectral index

## Abstract

**Background:**

To evaluate whether warm intranasal saline irrigation (38–40 °C) during functional endoscopic sinus surgery (FESS) is associated with reduced intraoperative bleeding compared with standard cold irrigation (18–21 °C).

**Methods:**

We performed a single-centre retrospective cohort study of consecutive FESS procedures under inhalational general anaesthesia (January 2017–December 2022). After exclusions, 1,990 cases were eligible. One-to-one propensity score matching (age, sex, ASA class, and type of inhaled anaesthetic) yielded 409 warm and 409 cold cases. Primary outcomes were estimated blood loss and blood loss >50 mL. Secondary outcomes included anaesthesia duration, intraoperative medication requirements, and lowest recorded temperature. Multivariable logistic regression adjusted for BIS monitoring utilisation, intravenous fluid rate, and intraoperative antihypertensive medication use.

**Results:**

Median blood loss was 50 mL (IQR 50–50) with warm irrigation versus 50 mL (IQR 50–100) with cold irrigation (*p* = 0.001). Blood loss >50 mL occurred in 18.6% versus 32.0%, respectively (*p* < 0.001). Warm irrigation remained independently associated with lower odds of blood loss >50 mL (adjusted OR 0.45, 95% CI 0.31–0.66; *p* < 0.001). BIS monitoring was also protective (adjusted OR 0.57, 95% CI 0.37–0.87; *p* = 0.009). Warm irrigation was associated with shorter anaesthesia duration (median 2.82 vs 3.38 h; *p* < 0.001) and a slightly higher nadir temperature (35.6 vs 35.5 °C; *p* = 0.007).

**Conclusions:**

Warm intranasal saline irrigation at 38–40 °C was associated with fewer clinically significant bleeding events during routine FESS and may represent a simple, low-cost adjunct to perioperative haemostatic management.

## Graphical abstract

**Figure:**
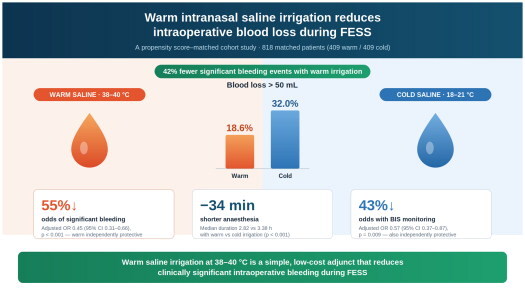

## Introduction

Functional endoscopic sinus surgery (FESS) represents the primary surgical intervention for chronic rhinosinusitis refractory to medical management, with intraoperative haemostasis remaining a critical determinant of surgical success and patient safety [1,2]. Inadequate control of intraoperative bleeding compromises endoscopic visualisation, potentially leading to prolonged operative time, incomplete disease clearance, and risk of complications including orbital injury and cerebrospinal fluid leak [3,4]. Even modest increases in mucosal bleeding can necessitate frequent instrument exchanges and irrigation cycles, substantially affecting workflow efficiency and potentially contributing to surgeon fatigue during lengthy procedures [2]. While various haemostatic strategies have been implemented in clinical practice, many carry inherent limitations including requirements for specialised anaesthetic expertise, risks of cardiovascular instability, or potential for tissue damage that may compromise postoperative healing and mucosal recovery [2]. Although absolute intraoperative blood loss during FESS is typically modest—often below 100 mL under optimised perioperative conditions—even small volumes of mucosal haemorrhage can meaningfully impair endoscopic visualisation and necessitate repeated field-clearing manoeuvres [5]. A blood loss threshold of 50 mL has accordingly been adopted as a clinically meaningful benchmark in the FESS literature, reflecting the volume above which surgical field clarity is detectably compromised and active haemostatic intervention becomes necessary [2,6,7].

Temperature modification of intranasal irrigation during FESS has been proposed as a simple, low-cost intervention requiring no specialised equipment beyond standard fluid warming capabilities [6,7]. Previous studies have reported that heated saline irrigation may improve surgical field conditions and reduce bleeding metrics, though most investigations utilised irrigation temperatures exceeding 45 °C and enrolled relatively small patient cohorts with limited follow-up duration [6,8]. A recent systematic review examining this intervention identified substantial heterogeneity in temperature protocols, irrigation volumes, and timing of application, as well as limited data addressing potential confounding by concurrent anaesthetic management practices that may independently affect bleeding [9]. Importantly, the comparative effectiveness of moderately warmed saline at near-physiologic temperatures versus standard cold irrigation remains inadequately characterised in large-scale clinical studies with rigorous control of confounding variables.

Several factors beyond irrigation temperature may independently influence intraoperative bleeding during FESS, creating challenges for causal inference in observational studies. Anaesthetic depth monitoring using bispectral index (BIS) has been associated with differences in haemodynamic stability, anaesthetic agent consumption, and recovery profiles, as documented in recent systematic reviews and perioperative studies examining various surgical populations [10–12]. The choice of volatile anaesthetic agent may affect vascular tone and bleeding propensity through differential effects on sympathetic activity and local tissue perfusion [13,14]. Additionally, fluid administration strategies during surgery influence intravascular volume status and may affect bleeding through haemodilution and alterations in coagulation factor concentrations, while requirements for antihypertensive medications during controlled hypotension vary substantially between patients and may confound comparisons of bleeding outcomes [15]. Concerns regarding perioperative hypothermia and its well-documented effects on coagulation factor activity and platelet function suggest that irrigation temperature may exert systemic physiologic effects extending beyond local haemostatic mechanisms [16].

Given the potential for confounding by indication and substantial practice variation in observational studies of perioperative interventions, propensity score matching has been advocated to balance measured covariates between treatment groups and improve causal inference in perioperative research when randomisation is not feasible [17–19]. However, large-scale propensity-matched analyses specifically evaluating moderately warmed irrigation in the range of 38–40 °C compared with cold irrigation at 18–21 °C during routine FESS practice are lacking in the published literature. Furthermore, the relationship between irrigation temperature and secondary perioperative parameters including total anaesthesia duration, intraoperative medication requirements, and core body temperature changes has not been comprehensively assessed in adequately powered studies.

The present study examined the association between warm intranasal saline irrigation (38–40 °C) versus cold saline irrigation (18–21 °C) and intraoperative bleeding during FESS in a large single-centre cohort treated at a tertiary referral hospital. Following propensity matching to create comparable groups, we used multivariable logistic regression analysis to adjust for residual differences in perioperative management factors including BIS monitoring utilisation, intravenous fluid administration rate, and antihypertensive medication use during the procedure. This analysis of clinical outcomes may inform the development of evidence-based perioperative protocols for FESS and identify patient populations most likely to benefit from this simple, accessible, and potentially cost-effective intervention.

## Methods

### Patient selection

This single-centre retrospective cohort study was conducted at Kaohsiung Chang Gung Memorial Hospital, Taiwan, in accordance with the Declaration of Helsinki, and approved by the Institutional Review Board of Kaohsiung Chang Gung Memorial Hospital (IRB number: 202301749B0). The requirement for informed consent was waived by the Institutional Review Board of Kaohsiung Chang Gung Memorial Hospital owing to the anonymised use of routinely collected data. Data collected between 1 January 2017 and 31 December 2022 were retrospectively analysed beginning in November 2023. We identified all consecutive patients who underwent FESS under general anaesthesia during this period. Cases were excluded if the procedure was not FESS, if inhalational anaesthesia was not used, or if records of inhaled anaesthetic consumption were incomplete. After these exclusions, 1,990 eligible cases remained, comprising 1,581 procedures in which cold saline irrigation was used and 409 procedures using warm saline irrigation.

**Figure 1.**
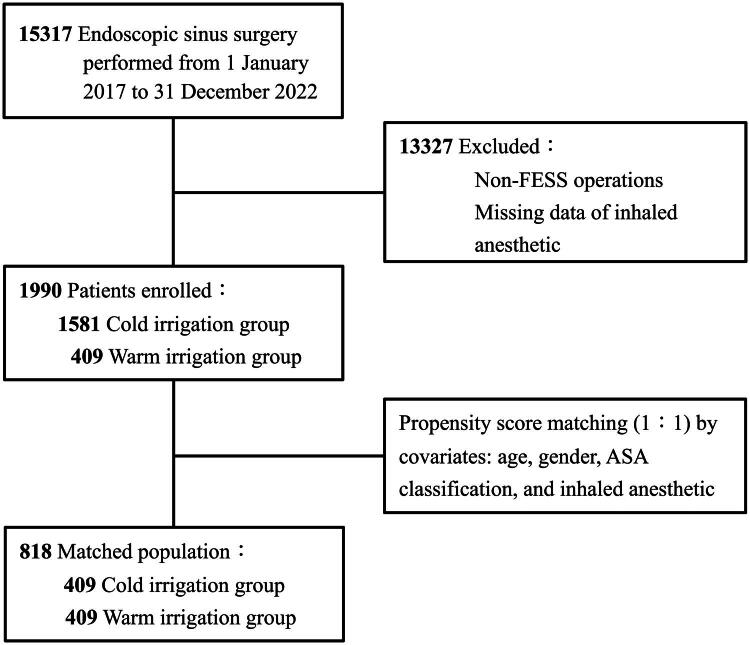
Patient selection flowchart. FESS procedures performed at Kaohsiung Chang Gung Memorial Hospital between January 2017 and December 2022 were screened for eligibility. After excluding non-FESS procedures and cases without inhalation anaesthesia or missing consumption records, 1,990 cases remained. Propensity score matching was performed based on gender, age, ASA classification, and type of inhalation agent (desflurane or sevoflurane), yielding 818 patients equally distributed between warm saline irrigation (*n* = 409) and cold saline irrigation (*n* = 409) groups. FESS, functional endoscopic sinus surgery; ASA, American Society of Anesthesiologists.

To reduce selection bias and balance baseline characteristics between groups, we performed 1:1 propensity score matching. Propensity scores were estimated using a logistic regression model including age, sex, American Society of Anesthesiologists (ASA) physical status classification, and type of inhaled anaesthetic (sevoflurane or desflurane). Nearest-neighbour matching without replacement and a fixed matching ratio of 1:1 were applied. This yielded a matched cohort of 818 patients, with 409 in the cold irrigation group and 409 in the warm irrigation group. The patient selection process is summarised in a flowchart (Figure 1).

### Irrigation preparation

Intranasal irrigation was performed using isotonic saline in both groups. In the cold irrigation group, saline was stored within the operating theatre at ambient room temperature (18–21 °C), reflecting standard practice in our institution. In the warm irrigation group, saline bags were placed in a thermostatically controlled water bath maintained at 38–40 °C and the temperature was confirmed immediately before use with an infrared thermometer. Throughout surgery, irrigation was delivered intermittently using 20-mL syringes by the first surgical assistant, as needed to clear blood and debris and optimise endoscopic visualisation. Because the first assistant prepared and administered the irrigation fluid, the operating surgeon—who was focused on the endoscopic dissection—and the patient, under general anaesthesia, were not aware of whether warm or cold saline was being used, reducing the potential for performance and observer bias in the assessment of bleeding. As this was a retrospective analysis of routinely collected data, irrigation temperature was not randomly assigned but reflected routine clinical practice and was ascertained from the operative and anaesthesia records; the study hypothesis was formulated only after these care episodes had occurred and therefore could not have influenced intraoperative management.

### Intraoperative procedure

General anaesthesia was induced with intravenous propofol (2–2.5 mg/kg), fentanyl (50–150 µg), and a neuromuscular blocking agent (cisatracurium 0.1–0.2 mg/kg or rocuronium 0.6–1.2 mg/kg) to facilitate tracheal intubation. Anaesthesia was maintained with either sevoflurane or desflurane in an oxygen/air mixture, chosen according to the attending anaesthesiologist’s preference. The minimum alveolar concentration of the inhaled agent was maintained between 0.6 and 1.2 to keep haemodynamic variables within 20% of baseline. In line with local reimbursement policy, bispectral index (BIS) monitoring was offered as an optional, self-paid service; when used, anaesthetic depth was titrated to a BIS value of 40–60. Nicardipine and labetalol were administered to treat episodes of intraoperative hypertension and maintain haemodynamic stability. All patients received active warming with forced-air warming blankets as part of routine perioperative temperature management.

Intraoperative monitoring followed standard institutional protocols and included continuous electrocardiography, non-invasive blood pressure, pulse oximetry, capnography, and temperature measurement using an axillary probe. Intravenous fluid administration, use and dosage of antihypertensive agents (nicardipine and labetalol), and inhaled anaesthetic consumption (expressed as mL/kg/hr) were recorded from the electronic anaesthesia information system. Total intraoperative blood loss was estimated by the surgical staff based on suction canister volume after subtraction of irrigation fluid and the number of blood-soaked surgical swabs.

### Outcome evaluation

The primary outcome measures were total intraoperative blood loss (mL) and the incidence of blood loss >50 mL during FESS. For regression analyses, blood loss was dichotomised as ≤50 mL versus >50 mL. This threshold reflects procedure-level blood loss volumes consistently reported in the FESS literature under optimised anaesthetic conditions, and corresponds to the volume above which clinically meaningful deterioration of surgical field quality has been documented [2,5]. Secondary outcomes included the intraoperative doses of nicardipine and labetalol, anaesthesia duration, consumption of inhaled anaesthetic agents (sevoflurane or desflurane, mL/kg/h), and the lowest recorded intraoperative body temperature (°C), as documented by the axillary temperature probe. Baseline characteristics and comorbidities, including hypertension, diabetes mellitus, prior cerebrovascular accident, and use of BIS monitoring, were collected to allow adjustment for potential confounders.

### Statistical analysis

All statistical analyses were performed after propensity score matching. Continuous variables are presented as median (interquartile range) because most distributions were non-normal on Kolmogorov-Smirnov testing. Categorical variables are summarised as numbers and percentages. Between-group comparisons were conducted using the Mann–Whitney *U* test for continuous variables and the chi-squared test or Fisher’s exact test, as appropriate, for categorical variables. To identify independent predictors of intraoperative blood loss >50 mL, we constructed univariate and multivariable logistic regression models using the matched cohort. Candidate covariates entered into the multivariable model included sex, age, BIS monitoring, irrigation temperature group (warm versus cold), hypertension, diabetes mellitus, type of inhaled anaesthetic (desflurane versus sevoflurane), inhaled anaesthetic consumption (mL/kg/h), intravenous fluid administration rate (mL/kg/h), nicardipine dose, labetalol dose, and lowest intraoperative body temperature. Results are reported as odds ratios (ORs) with 95% confidence intervals (CIs). A two-sided *p* value <0.05 was considered statistically significant. All analyses were performed using standard statistical software. To address the potential influence of revision surgery, we performed two sensitivity analyses: (1) refitting the multivariable model with revision status (primary versus revision FESS) added as an additional covariate, and (2) restricting the analysis to primary FESS cases. Because this was a retrospective analysis of a fixed matched cohort, an a priori sample-size calculation was not applicable; we therefore performed a sensitivity (minimum detectable effect) analysis for the primary categorical outcome at a two-sided α of 0.05.

## Results

After propensity score matching, a total of 818 patients were included in the final analysis, with 409 patients assigned to the cold saline irrigation group and 409 to the warm saline irrigation group (Figure 1). Baseline demographic characteristics and clinical profiles were well balanced between groups after matching (Table 1). There were no statistically significant differences in age, sex distribution, body weight, ASA physical status classification, or major comorbidities, including hypertension, diabetes mellitus, and prior cerebrovascular accident. The median age in both groups was 47 years, and the majority of patients were classified as ASA II. Intravenous fluid administration rates were also comparable between groups. However, the proportion of cases in which bispectral index (BIS) monitoring was applied was higher in the warm irrigation group than in the cold irrigation group (39.1% vs. 22.7%, *p* < 0.001).

**Table 1.** Baseline characteristics and patient demographics after propensity score matching.

Variables (unit)	n (%)/ median (IQR)	Cold irrigation group (*n* = 409)	Warm irrigation group (*n* = 409)	*p value*
Gender				0.292
Female	369 (45.1%)	177 (43.3%)	192 (46.9%)	
Male	449 (54.9%)	232 (56.7%)	217 (53.1%)	
Age (years)	47.0 (36.0–58.0)	47.0 (35.0–58.0)	47.0 (36.0–58.0)	0.755
Body weight (kg)	66.0 (56.0–77.0)	65.0 (56.5–78.0)	66.0 (56.0–76.0)	0.684
ASA physical status				0.697
I	106 (13.0%)	56 (13.7%)	50 (12.2%)	
II	618 (75.6%)	309 (75.6%)	309 (75.6%)	
III & IV	94 (11.5%)	44 (10.8%)	50 (12.2%)	
Hypertension	167 (20.4%)	86 (21.0%)	81 (19.8%)	0.665
Diabetes mellitus	77 (9.4%)	40 (9.8%)	37 (9.0%)	0.719
Cerebrovascular accident	12 (1.5%)	6 (1.5%)	6 (1.5%)	1.000
Bispectral index applied	253 (30.9%)	93 (22.7%)	160 (39.1%)	<0.001*
Rocuronium and sugammadex	51 (6.2%)	26 (6.4%)	25 (6.1%)	0.885
Intravenous fluid (mL/kg/h)	2.36 (1.89–2.98)	2.33 (1.89–2.99)	2.39 (1.89–2.98)	0.511

Note: Data are presented as n (%) or median (interquartile range). ASA, American Society of Anesthesiologists; BIS, bispectral index. *Statistically significant (*p* < 0.05). Propensity score matching was performed based on gender, age, ASA classification, and inhalation agent type. Statistical analyses: Kolmogorov-Smirnov test for normality assessment, Mann-Whitney *U* test for continuous variables, chi-squared test or Fisher’s exact test for categorical variables.

### Primary outcomes

Intraoperative blood loss was significantly lower in the warm saline irrigation group compared with the cold irrigation group (Table 2). Median blood loss was 50 mL (interquartile range [IQR] 50–50) in the warm irrigation group versus 50 mL (IQR 50–100) in the cold irrigation group (*p* = 0.001). When blood loss was analysed categorically, a significantly higher proportion of patients in the warm irrigation group experienced blood loss ≤50 mL compared with those in the cold irrigation group (81.4% vs. 68.0%), whereas blood loss >50 mL occurred less frequently in the warm irrigation group (18.6% vs. 32.0%; *p* < 0.001). With 409 patients per group, the study had 80% power to detect an absolute between-group difference in the incidence of blood loss >50 mL of approximately 9 percentage points; the observed difference (18.6% vs 32.0%) exceeded this threshold, indicating that the sample size was adequate for the primary comparison. The corresponding achieved (post-hoc) power for the observed effect was high (>99%) but is reported for transparency only and interpreted with caution, as post-hoc power is a deterministic function of the observed result.

**Table 2.** Primary and secondary outcomes.

Variables (unit)	*n* (%)/median (IQR)	Cold irrigation group (*n* = 409)	Warm irrigation group (*n* = 409)	*p value*
Blood loss (mL)	50 (50–60)	50 (50–100)	50 (50–50)	0.001*
Blood loss categories				<0.001*
≤50 mL	611 (74.7%)	278 (68.0%)	333 (81.4%)	
>50 mL	207 (25.3%)	131 (32.0%)	76 (18.6%)	
Antihypertensive agent used				
Nicardipine dosage (mg)	0.3 (0.0–1.0)	0.0 (0.0–0.6)	0.5 (0.0–1.0)	<0.001*
Labetalol dosage (mg)	0.0 (0.0–2.5)	0.0 (0.0–2.5)	1.0 (0.0–2.5)	<0.001*
Anaesthesia duration (hr)	3.08 (2.45–3.83)	3.38 (2.67–4.17)	2.82 (2.25–3.49)	<0.001*
Sevoflurane dosage (mL/kg/h)	0.22 (0.18–0.26)	0.20 (0.17–0.25)	0.23 (0.20–0.28)	<0.001*
Desflurane dosage (mL/kg/h)	0.33 (0.26–0.41)	0.33 (0.25–0.36)	0.35 (0.27–0.43)	0.156
Lowest body temperature (°C)	35.6 (35.2–35.9)	35.5 (35.1–35.9)	35.6 (35.3–36.0)	0.007*

Note: Data are presented as n (%) or median (interquartile range). *Statistically significant (*p* < 0.05). Primary outcomes: intraoperative blood loss and proportion of patients with blood loss >50 mL. Secondary outcomes: antihypertensive agent dosage (nicardipine and labetalol), anaesthesia duration, inhaled anaesthetic consumption (sevoflurane or desflurane), and lowest intraoperative body temperature. Statistical analyses: Kolmogorov-Smirnov test for normality assessment, Mann-Whitney *U* test for continuous variables, chi-squared test for categorical variables.

### Secondary outcomes

Several secondary intraoperative outcomes differed significantly between groups (Table 2). The warm irrigation group required higher doses of antihypertensive agents during surgery, including both nicardipine (median 0.5 mg [IQR 0.0–1.0] vs. 0.0 mg [IQR 0.0–0.6], *p* < 0.001) and labetalol (median 1.0 mg [IQR 0.0–2.5] vs. 0.0 mg [IQR 0.0–2.5], *p* < 0.001), compared with the cold irrigation group.

Anaesthesia duration was significantly shorter in the warm irrigation group, with a median duration of 2.82 h (IQR 2.25–3.49), compared with 3.38 h (IQR 2.67–4.17) in the cold irrigation group (*p* < 0.001). Median operative (surgical) duration was also shorter in the warm irrigation group (2.07 h, IQR 1.57–2.77) than in the cold irrigation group (2.62 h, IQR 1.98–3.38; *p* < 0.001). The median total intraoperative crystalloid volume was 450 mL (IQR 300–600) in the warm group versus 500 mL (IQR 350–750) in the cold group (*p* < 0.001), although weight- and time-indexed infusion rates were comparable between groups (2.39 vs 2.33 mL/kg/h; *p* = 0.55). Regarding inhaled anaesthetic consumption, patients receiving warm irrigation demonstrated higher sevoflurane consumption (median 0.23 vs. 0.20 mL/kg/hr, *p* < 0.001), whereas desflurane consumption did not differ significantly between groups. The lowest recorded intraoperative body temperature was slightly but significantly higher in the warm irrigation group (median 35.6 °C vs. 35.5 °C, *p* = 0.007).

### Regression analyses

Univariate and multivariable logistic regression analyses were performed to identify independent predictors of intraoperative blood loss >50 mL (Table 3). In univariate analysis, male sex, a higher intravenous fluid administration rate, and labetalol dosage were significantly associated with increased odds of blood loss >50 mL, whereas increasing age, BIS monitoring, and warm saline irrigation were significantly associated with reduced odds. In the multivariable model, warm saline irrigation remained independently associated with a significantly lower risk of blood loss >50 mL (adjusted odds ratio [OR] 0.45, 95% confidence interval [CI] 0.31–0.66; *p* < 0.001). BIS monitoring was also independently protective (adjusted OR 0.57, 95% CI 0.37–0.87; *p* = 0.009).

**Table 3.** Univariate and multivariable logistic regression analysis for intraoperative blood loss >50 mL.

Variables (unit)	*n* (%)	Univariate analysis		Multivariable analysis	
		OR (95% CI)	*p* value	OR (95% CI)	*p* value
Female	369 (45.1%)	1 (Reference)		1 (Reference)	
Male	449 (54.9%)	2.16 (1.55–3.02)	<0.001*	2.10 (1.43–3.07)	<0.001*
Age	47.0 (36.0–58.0)	0.98 (0.97–0.99)	0.001*	0.98 (0.97–1.00)	0.027*
BIS applied	253 (30.9%)	0.58 (0.40–0.83)	0.003*	0.57 (0.37–0.87)	0.009*
Cold saline irrigation	409 (50.0%)	1 (Reference)		1 (Reference)	
Warm saline irrigation	409 (50.0%)	0.48 (0.35–0.67)	<0.001*	0.45 (0.31–0.66)	<0.001*
Hypertension	167 (20.4%)	0.91 (0.61–1.36)	0.652	1.21 (0.72–2.03)	0.465
Diabetes mellitus	77 (9.4%)	0.96 (0.56–1.66)	0.894	1.27 (0.67–2.39)	0.465
Sevoflurane inhaled	753 (92.1%)	1 (Reference)		1 (Reference)	
Desflurane inhaled	65 (7.9%)	1.57 (0.92–2.90)	0.101	2.03 (1.04–3.96)	0.038*
Inhaled anaesthetic (mL/kg/h)	0.22 (0.18–0.27)	0.79 (0.11–5.72)	0.811	0.34 (0.03–3.61)	0.368
Intravenous fluid (mL/kg/h)	2.36 (1.89–2.98)	1.24 (1.05–1.47)	0.013*	1.47 (1.20–1.81)	<0.001*
Nicardipine dosage (mg)	0.3 (0.0–1.0)	1.14 (0.97–1.33)	0.115	1.12 (0.92–1.37)	0.246
Labetalol dosage (mg)	0.0 (0.0–2.5)	1.06 (1.00–1.12)	0.036*	1.04 (0.98–1.12)	0.212
Lowest body temperature (°C)	35.6 (35.2–35.9)	1.17 (0.96–1.60)	0.316	1.41 (0.98–2.04)	0.066

Note: OR, odds ratio; CI, confidence interval; BIS, bispectral index. *Statistically significant (*p* < 0.05). The outcome variable was dichotomised with blood loss ≤50 mL coded as ‘0’ (reference) and blood loss >50 mL coded as ‘1’. Multivariable analysis included all variables from univariate analysis. Variables independently associated with increased odds of blood loss >50 mL (OR >1.0) were male sex (reference: female), desflurane use (reference: sevoflurane), and a higher intravenous fluid administration rate. Variables associated with reduced odds of blood loss >50 mL (OR <1.0) were increasing age, BIS monitoring application, and warm saline irrigation (reference: cold saline).

Male sex was associated with higher odds of blood loss >50 mL compared with female sex (adjusted OR 2.10, 95% CI 1.43–3.07; *p* < 0.001), while increasing age was associated with a modest reduction in risk (adjusted OR per year 0.98, 95% CI 0.97–1.00; *p* = 0.027). Use of desflurane, compared with sevoflurane, was independently associated with increased odds of blood loss >50 mL (adjusted OR 2.03, 95% CI 1.04–3.96; *p* = 0.038). Higher intravenous fluid administration rates were also independently associated with increased blood loss (adjusted OR 1.47 per mL/kg/h, 95% CI 1.20–1.81; *p* < 0.001). Hypertension, diabetes mellitus, nicardipine dose, inhaled anaesthetic consumption, and the lowest intraoperative body temperature were not significantly associated with blood loss >50 mL in either univariate or multivariable analysis. Labetalol dose was significantly associated with increased odds of blood loss >50 mL in univariate analysis (OR 1.06, 95% CI 1.00–1.12; *p* = 0.036) but was no longer significant after multivariable adjustment (OR 1.04, 95% CI 0.98–1.12; *p* = 0.212).

Revision surgery was uncommon (35/818, 4.3%) and occurred more frequently in the warm irrigation group (29 [7.1%] vs 6 [1.5%]). In a sensitivity analysis that added revision status to the multivariable model, the association between warm irrigation and blood loss >50 mL was unchanged (adjusted OR 0.44, 95% CI 0.30–0.65; *p* < 0.001), and revision surgery itself was not independently associated with bleeding (adjusted OR 1.04, 95% CI 0.42–2.59; *p* = 0.94). When the analysis was restricted to primary FESS cases (*n* = 783), warm irrigation remained independently associated with lower odds of blood loss >50 mL (adjusted OR 0.45, 95% CI 0.30–0.66; *p* < 0.001).

## Discussion

Warm intranasal saline irrigation at near-physiological temperature was associated with a lower incidence of clinically significant intraoperative bleeding during FESS in this propensity score-matched cohort. Although median estimated blood loss was low in both groups, warm irrigation was associated with a markedly reduced probability of blood loss exceeding 50 mL, and this association remained robust after multivariable adjustment. Even modest reductions in diffuse mucosal oozing can meaningfully improve endoscopic visualisation, and thereby facilitate operative efficiency and reduce the need for repeated field clearing. These findings support irrigation temperature as a pragmatic, modifiable perioperative factor that may contribute to optimised surgical field conditions in routine endoscopic sinonasal surgery.

The haemostatic benefit of warm or heated saline irrigation has been reported in randomised trials and systematic reviews of endoscopic sinus surgery, with consistent evidence supporting improved bleeding control and surgical field visibility without major safety concerns [8,9,20]. Our findings extend this literature by demonstrating that a moderate temperature range (38–40 °C), closer to physiological conditions, is associated with meaningful reductions in clinically relevant bleeding in routine practice. This temperature range may enhance clinical acceptability by minimising concerns regarding mucosal thermal injury while preserving haemostatic benefit [7]. Moreover, a categorical threshold of blood loss >50 mL may better capture clinically relevant differences than median blood loss alone when overall blood loss is clustered at low volumes, as commonly observed during FESS. This dichotomy is further grounded in quantitative studies of routine FESS under controlled anaesthetic conditions, which have reported procedure-level blood loss in the range of 40–70 mL, directly bracketing the 50 mL threshold and confirming its empirical relevance as a clinical endpoint [5,21].

Several mechanisms may explain the association between irrigation temperature and bleeding. Local thermal effects on sinonasal mucosa may modulate microvascular tone and reduce capillary oozing, which constitutes a dominant source of bleeding during FESS [6,9]. Small changes in capillary ooze can disproportionately influence endoscopic visibility and the frequency of haemostatic manoeuvres. In addition, irrigation fluids contribute to perioperative heat balance, and perioperative hypothermia, even when mild, has been shown to impair platelet function and coagulation efficiency [22]. Although the absolute difference in lowest recorded intraoperative temperature between groups was small, nadir temperature may underestimate cumulative thermal burden. Warmer irrigation may reduce episodic mucosal cooling and overall thermal stress, effects not fully captured by a single temperature measurement [16].

An important observation was the higher use of antihypertensive agents for intraoperative haemodynamic management in the warm irrigation group despite lower bleeding. This finding argues against a simplistic explanation that reduced blood loss was achieved solely through more intensive controlled hypotension. While controlled hypotension is frequently used to improve surgical field conditions in FESS, contemporary perioperative care emphasises patient safety and individualised haemodynamic targets rather than routine deep hypotension [23,24]. Observational data further suggest that hypotensive strategies and bleeding outcomes are influenced by multiple co-interventions, including anaesthetic depth, ventilation, vasoconstrictor use, and fluid management [25]. In our study, antihypertensive agents were administered primarily to treat episodes of intraoperative hypertension rather than to deliberately induce controlled hypotension. In multivariable analysis, antihypertensive use was not independently associated with surgical blood loss. The dissociation between antihypertensive use and bleeding reduction supports the hypothesis that local effects of irrigation temperature contribute substantially to haemostasis beyond systemic blood pressure modulation alone.

Bispectral index (BIS) monitoring emerged as an independent protective factor against blood loss exceeding 50 mL. Evidence indicates that BIS-guided anaesthesia facilitates more precise titration of anaesthetic depth, reduces anaesthetic consumption, and improves early recovery outcomes [11,26], with supportive data in perioperative contexts relevant to recovery quality [27]. In the present study, BIS monitoring remained imbalanced after propensity score matching, reflecting its availability as a self-paid service and potential association with more protocolised anaesthetic management. This residual imbalance represents an important limitation because BIS use may act as a surrogate for a broader bundle of care practices. Nevertheless, multivariable regression demonstrated that both warm irrigation and BIS monitoring independently contributed to reduced bleeding risk, suggesting that their effects are not entirely collinear and that the association with warm irrigation persists after accounting for BIS use [18].

Male sex was independently associated with increased intraoperative bleeding, whereas increasing age showed a modest protective effect. Sex-related differences in haemostasis and vascular physiology have been described, including clinically relevant differences in thrombosis and haemostatic regulation [28,29]. Consistent with this biological plausibility, surgical cohorts have reported sex-associated differences in haemostatic parameters and perioperative bleeding outcomes [30]. Age-related vascular changes, including reduced microvascular density and altered inflammatory responses, may partly explain the inverse association between age and bleeding risk observed in this cohort [31].

From an anaesthetic perspective, desflurane use was associated with higher odds of clinically significant bleeding compared with sevoflurane. Volatile anaesthetics differ in their effects on sympathetic tone and vasodilation, and sevoflurane has been suggested to provide more stable haemodynamic conditions in some surgical settings [32]. However, volatile choice may also reflect clinician preference and case characteristics; therefore, this finding should be considered hypothesis-generating. Higher intravenous fluid administration rates were also independently associated with increased bleeding risk. The crystalloid volumes administered in this cohort were modest, however (median 450–500 mL, ∼9%–11% of estimated blood volume), and far below the degree of haemodilution—generally exceeding 60% of circulating blood volume—required to produce clinically significant dilutional coagulopathy. This association therefore most plausibly reflects local haemodynamic effects, such as mucosal oedema and increased venous and capillary pressure promoting diffuse field oozing, and/or reverse causation, whereby greater bleeding prompts more liberal fluid replacement, rather than systemic dilution of coagulation factors [33–35]. Notably, weight- and time-indexed fluid rates were comparable between groups, and warm irrigation remained independently protective after adjustment for fluid rate, indicating that this association does not account for the primary finding, underscoring the importance of judicious fluid management during FESS.

Warm irrigation was additionally associated with shorter anaesthesia duration, consistent with reports that improved surgical field visibility reduces interruptions related to bleeding control [6,7]. However, reverse causality cannot be excluded, as shorter procedures may involve less cumulative irrigation and thermal exposure. Operative duration is influenced by disease severity, surgical extent, and surgeon experience—factors not fully captured in this retrospective dataset [36].

This study has limitations. Its retrospective, single-centre design precludes definitive causal inference despite rigorous propensity score matching and multivariable adjustment [18,37]. Estimated blood loss is semi-quantitative and subject to measurement variability, particularly in procedures involving substantial irrigation [38]. Residual confounding remains possible, especially given the imbalance in BIS monitoring and the absence of standardised measures of disease severity such as polyp burden or radiographic staging [39]. Furthermore, although prior sinus surgery is a recognised predictor of poorer intraoperative surgical-field conditions and greater bleeding [21], our sensitivity analyses indicated that the warm-irrigation association was not driven by revision surgery; nevertheless, revision cases were few (*n* = 35), and together with the lack of disease-severity staging noted above, residual confounding by baseline bleeding risk cannot be entirely excluded.

In conclusion, warm intranasal saline irrigation at 38–40 °C was associated with a lower risk of clinically significant intraoperative bleeding during FESS in a propensity score–matched cohort. This simple, low-cost intervention may serve as a pragmatic adjunct to contemporary perioperative management strategies, complementing optimised anaesthetic depth control and fluid management within enhanced recovery–oriented care pathways [5,21,40]. Prospective multicentre studies are warranted to confirm these findings and define standardised irrigation protocols within evidence-based perioperative pathways.

## Data Availability

The data supporting the findings of this study are available from the corresponding author (S.C.W.) upon reasonable request and subject to institutional data-sharing policies.

## Abbreviations

ASA: American Society of Anesthesiologists
BIS: bispectral index
CI: confidence interval
FESS: functional endoscopic sinus surgery
h: hour
IQR: interquartile range
IRB: Institutional Review Board
kg: kilogram
mL: millilitre
OR: odds ratio
